# Simulation-based education as a provider of fieldwork insights – experiences of ambulance nurse specialist students

**DOI:** 10.1186/s12912-023-01666-2

**Published:** 2023-12-19

**Authors:** Ulf Andersson, Gabriella Norberg Boysen, Anders Sterner

**Affiliations:** 1 Centre for Prehospital Research, Faculty of Caring Science, Work Life and Social Welfare, University of Borås, Borås, SE-501 90 Sweden; 2 Faculty of Caring Sciences, Work Life and Social Welfare, University of Borås, Borås, 501 90 Sweden

**Keywords:** Simulation-based education, Survey Research, Content analysis, Education, Ambulance, Specialist nursing

## Abstract

**Background:**

Medicine is facing a global shortage of nurses, including those with postgraduate education. One suggested educational method for undergraduate and postgraduate education, such as specialist ambulance nurse education, is simulation-based education (SBE). The implementation of SBE is motivated, in part, by the desire to attract and retain students, but also to contribute to student learning. Consequently, the use of SBE is increasing in specialist ambulance nurse education. The aim of this study was to explore how specialist ambulance nursing students experience SBE.

**Methods:**

This qualitative survey study involved the collection of study data using a purposefully designed, paper-based survey comprising five open-ended questions that required participant free-text answers. The answers were analysed using inductive content analysis and searching for descriptions of the participants’ experiences. The survey was presented to 35 specialist ambulance nursing students.

**Results:**

The results are presented in two themes: *SBE as learning* and *SBE as an educational method*. Participating in SBE during the programme provides students with a realistic understanding of their future profession and its expected demands. The learning experience disregards prior work experience in ambulance services.

**Conclusions:**

Based on the findings, conclusions are that SBE is an appreciated educational method among nursing students, regardless of their prior experience in the field of prehospital care. To some extent, this differs from previous research findings related to this subject. Furthermore, SBE contributes to the provision of field work insights, preparing the ambulance nurse specialist students.

**Supplementary Information:**

The online version contains supplementary material available at 10.1186/s12912-023-01666-2.

## Background

Registered nurses (RNs) are often the first health professionals that people meet; therefore, RNs are essential in health promotion, disease prevention and provision of primary, community and emergency care. However, the World Health Organization reports a global shortage of RNs [1], and Sweden is no exception [2].

One reason for the shortage of RNs is the nursing student attrition rate, as determined by numerous studies [3]. Strategies for increasing retention require an understanding of students’ motivations to become RNs [4], and this need has led to several studies exploring this area. A review of the factors influencing an RN career choice showed strong intrinsic elements, such as a motivation to help others and a personal interest in healthcare. External factors included job security, but other factors, such as family, financial remuneration and professional prestige, had only weak or inconclusive influences [5]. One suggestion made to attract potential students to the nursing field is the use of simulation and gaming [6]. Successful strategies that can increase nursing student retention include peer mentorship, discussions with mentors and clinical advisors, and a focus on active learning, which includes simulation-based education (SBE) [7].

RNs with nurse specialist education are in considerably short supply, and this shortage is expected to persist or increase up to 2035 [2]. Nurse specialist education improves patient health outcomes and assists in meeting the emerging specialisation of nursing practice [8]. In Sweden, the requirements for becoming an RN specialist include completion of a 3 year (180 European Credit Transfer System [ECT]) bachelor’s degree in nurse education and 1 year of additional post-graduate education. After completion, the RN becomes a specialist and receives, for example, a postgraduate diploma in prehospital emergency care and a master’s degree with a major in nursing or caring science (60 ECTS) [9]. Upon receipt of the diploma and degree, the RN can gain the title of a clinical nurse specialist in prehospital emergency care (henceforth called the specialist ambulance nurse [SAN]).

In Sweden, nurse specialist education is not officially required to work in prehospital emergency care (i.e. ambulance). Thus, ambulance personnel could consist of emergency medical technicians, an RN or a nurse specialist, such as SAN, and/or a physician with or without a specialisation. The various roles can be related to the level of care provision of basic life support. For example, technicians can provide basic CPR, fracture splinting and, in some cases, oxygen administration, whereas RNs and SAN provide advanced life support, including invasive procedures, such as endotracheal intubation and intravenous lines, and administration of potent controlled drug [10]. However, since 2005, each ambulance crew must contain at least an RN [11]. Recently, some of the 21 regions of Sweden responsible for ambulance services have independently started to require nurse specialists to have a degree for permanent employment [12]. Nonetheless, SAN has a competence description that identifies, clarifies the professional knowledge, competence and responsibility needed for the development of care in the prehospital context [13].

The ambulance system utilised in Sweden is a combination of the Anglo-American and Franco-German system [14]. This means that the patient could either be rushed to the emergency department or, based on an RN or physician assessment, could be bypassed to specialist care, referred to the local healthcare centre, or left at the scene with self-care advice [15]. However, a direct comparison between the standards for care provision and the ambulance system is difficult, as no unified measurements are available [10].

### Simulation-based education

SBE can be described as a technique that creates a situation in which participants can experience a representation of a real event as a way to practice, learn, evaluate, test and understand systems (i.e. healthcare processes) or human actions [16]. One frequently cited learning theory in the nursing-related SBE literature is Kolb’s theory of experiential learning. This theory emphasises the role of experience in learning, wherein learning becomes an adaptive process in which experiences are transformed into knowledge. Reflective dialogue, observation and/or active experimentation enable the individual to grasp an experience and transform it into knowledge [17].

In undergraduate nursing education, SBE is a popular method of teaching and learning. It has been found to contribute to student learning and improved knowledge, while also enhancing clinical skills acquisition, self-efficacy, confidence and competence [18]. SBE has been increasingly used in postgraduate nursing education [19], as well as serving as a measure for continued professional development among RNs [20]. Wheeler and Dippenaar [21], in their review on this topic, conclude that SBE is the primary measure for teaching and training paramedic students. Research in the area of SBE and paramedicine has evolved to explore the use of and evidence for specific methods, such as augmented reality and mixed reality modalities [22], as well as to compare the effectiveness of these methods to conventional teaching [23]. However, to our knowledge, little is known about using SBE as a measure to teach and develop knowledge and competence among RNs during a specialist ambulance nursing programme.

In summary, SBE is a commonly used educational method in different nursing education programmes. Even so, a knowledge gap remains regarding the experiences of SAN students with SBE. Consequently, exploring the experiences of SAN students is necessary to gain knowledge about the influence of SBE on these students. The findings could contribute to the development of SBE geared towards SAN programmes as well as other postgraduate nursing educational programmes.

## Methods

### Aim

The aim of this study was to describe SAN students’ experiences with SBE.

### Design

An inductive qualitative and exploratory research design was adopted using a paper-based survey method with free text answers [24]. The study also followed the reporting standards of the Enhancing the QUAlity and Transparency Of health Research (EQUATOR) [25] and the Standards for Reporting Qualitative Research checklist [25]. The description of the setting and SBE were inspired by the key elements used to report simulation-based research [26].

### Setting

This study was conducted at a university simulation centre in the southwest region of Sweden. The centre provides high-fidelity SBE experiences for diverse undergraduate students and postgraduate healthcare education programmes. All personnel (operators and facilitators) have completed a course to be medical simulation instructors and are accustomed to this pedagogical teaching method (3–8 years) and the SBE equipment utilised. In addition, they are RN specialists with extensive experience (≥ 5–20 years) in ambulance or emergency care (i.e. emergency department and intensive care).

### Preparation of the simulation-based education

The equipment utilised is similar to that used in clinical work, apart from the pharmaceuticals and the reuse of otherwise disposable and expensive consumables. The simulator or embedded patient is placed in a position appropriate to the scenario, using realistic obstructions or space limitations (i.e. furniture, rugs). The SBE was conducted over a three-day period, with three different scenarios (A, B, C. Table 1) on each day. The SAN student took part in one of these scenarios on each day. The instructors were responsible for one of the scenarios during the three days to ensure a standardised approach. Each scenario was played out twice, but with some difference between them (see the scenario description further down). This variation was meant to challenge the students in the second scenario version to another approach to the situation. The SAN students were either active in patient care or observed from another room (via video), and these roles were switched between the two versions of the scenario.

#### The scenarios

Personnel from the SAN programme and simulation centre developed the SBE scenarios (A, B, C. Table 1), which was based on the recommendations of the International Nursing Association of Clinical and Simulation Learning (INACSL) [27]. The scenarios were scripted in terms of the changing vital signs and progression of the patient’s condition. However, these changes were realistically adapted based on the interventions conducted by the SAN students. The possibility was left open for the facilitator to provide information that was either requested from the students or deemed necessary for the continuation of the scenario.

All scenarios involve the hybrid combination of a manikin (Gaumard – HAL 3101, and Gaumard - pediatric HAL S3004) and an embedded participant (instructor). The manikins allowed assessment of the pulse, blood pressure, heart sounds, pupil reaction, breathing sounds, chest movements, swelling of the tongue, cyanosis and motor skills of the eyes. Limitations included the restriction of movement of limbs, lagging audio transfer for voice, and the inability to administer intravenous lines and drugs.

Scenario A1 involves a 1.5-year-old with a fever convulsion. The child is attended by a worried parent. The scene takes place in the environment of a tidy, one-room apartment. The apartment is furnished with a crib, toys, an armchair, a rug and a small table with two chairs.

Scenario A2 involves a 1.5-year-old with a recent head injury and fever convulsion. The child is attended by a distanced parent. The scene takes place in the environment of an apartment with signs of substance abuse. The apartment is furnished essentially the same as in scenario A1, but with the addition of a used syringe containing an unknown substance, pill blisters with tramadol, beer cans, a wine bottle and some cigarette butts.

Scenario B1 involves a 60-year-old male who has experienced a myocardial infarction/cardiac arrest. The scene takes place in a local shopping mall. Scenario B2 involves a 20-year-old male who has experienced anaphylactic shock. The man is attended by a personal assistant. The scene takes place in a local shopping mall. These scenarios were furnished with two shopping cart trolleys with various food packages. Also included is a food stand with a doll serving taste-portions of a new food product, as well as a loudspeaker broadcasting various messages to the store customers.

Scenario C1 involves a 78-year-old male with stroke. The scene takes place in an apartment in which the man lives alone. Some language barriers exist. Scenario C2 involves a 78-year-old male with a traumatic head injury. The scene takes place in an apartment. Again, some language barriers are evident. The apartment, in both scenario versions, is furnished with an armchair and a small coffee table with a coffee cup and a potted plant, a newspaper stand, a rug and a bed.

**Table 1:** Scenario overview

Scenario	Chief complaint	Patient (Manikin/Embedded participant)	Additional roles (Embedded participant)	Environment
A1 A2	Fever convulsion Head injury and convulsion	Manikin Manikin	Embedded participant Embedded participant	Apartment (tidy) Apartment (harsh)
B1 B2	Myocardial infarction – Cardiac arrest Anaphylactic shock	Manikin Manikin	None Embedded participant	Shopping mall Shopping mall
C1 C2	Stroke Head trauma	Embedded Embedded	None None	Apartment Apartment

### Procedure before and during the SBE day

In line with INACSL recommendations, all simulations used four phases: pre-briefing, briefing, scenario and debriefing [27].

The pre-briefing was performed through a series of steps: (1) On the introduction day (12–15 weeks prior to study) of the SAN programme, information was provided explaining that SBE is used as a pedagogical method and describing the learning objectives associated with it. (2) During the introduction to the specific course, the learning objectives for the specific SBE were presented. These learning objectives are also presented in the course curriculum. (3) The study guide for the course contains descriptions of what is needed in terms of receiving a pass/fail grade on the course (i.e. to receive a pass grade, the student needed to have actively participated in SBE). (4) The SAN students were provided with an SBE manual that informed them that the SBE would involve three scenarios related to patient conditions, such as convulsions, unconsciousness and unspecified patient information. (5) In summary, the pre-briefing consisted of approximately 1.5 h of verbal information, with the possibility for the students to read on their own. At the beginning of the SBE day, the students were paired and allocated to their specific scenarios for the day. The information from the previous steps was summarised into the students’ learning objectives for the day: to assess the patients’ condition and, based on the assessment, to initiate, conduct and evaluate interventions and care that the patient’s condition requires.

The briefing session for each specific scenario (A, B, C) involved the limitations for the upcoming scenario (i.e. what could or could not be done with the manikin/embedded patient, and procedures for calling additional resources). The SAN students were also encouraged to check their ambulance equipment (i.e. a backpack/bag with equipment for measuring vital signs, drugs for administration, paper journals, and a stretcher). Immediately before the start of the scenario, the students were provided with information regarding the current weather and distance to hospital, along with dispatch information regarding the specific case (priority, address, gender, age, medical history and the current issue). The SAN students who would encounter the patient were allowed to discuss strategy. The observers were charged with a task to which they were to pay special attention during the SBE (i.e. the nature of the communication within the team). The briefing session lasted approximately 5–10 min.

The scenarios (A, B and C) were played out twice, with some differences. The pairs of SAN students assigned to the scenario changed roles from active to observing and vice versa between the two versions. During the scenario, the students were expected to assess the situation, examine the patient (manikin/embedded participant), provide suitable treatment and make decisions. Each scenario lasted approximately 20–25 min. A debriefing was held after each scenario.

The debriefing session was conducted immediately after each scenario and was based on a modified version of “The diamond” debriefing model [28]. The modification involved an additional aspect of reflection on the scenario and the performance in relation to course literature, lectures or research. The SAN students were asked to share their thoughts, feelings and experiences regarding the SBE scenario itself, or what they would take with them into their future profession. The debriefing session lasted approximately 50 min.

### Participants

The participants in this study were students enrolled in a mandatory course in the SAN programme; thus, all students were in the same stage of their education. They received written and verbal information about the study and were instructed that participation was voluntary and that the choice of participation would not influence their grades during the specific programme. Verbal information included the possibility of participating in a research study regarding SBE and that participation involved answering a paper-based survey. The verbal information was provided on the introduction day of the SAN programme, in the previous course, and at the beginning of the SBE day. Written information was provided after the debriefing session on the SBE day, together with the paper-based survey and the request for participation.

The SAN students participating in this study had prior experience with SBE from an earlier course in the SAN programme. The experience included an introduction to SBE (1.5 h, 6–8 weeks prior to the study), in which SAN students were introduced and oriented to the equipment utilised and its functions and limitations. The students had the opportunity to try these functions themselves (e.g. listening to chest sounds, feeling for pulses). Three SBE sessions (5 weeks prior to the study) were also held in which the SAN students assessed their patients using the A-E-principle according to the course concept of advanced medical life support [29]. During these SBE sessions, the SAN students also obtained experience from the various briefing sessions utilised. These prior experiences in the SAN programme had familiarised and oriented the students to the equipment and procedures used in the SBE before they attended the SBE days that were the focus of this study.

### Data collection

The paper-based survey was presented to the SAN students after each SBE day so that each student had the opportunity to provide answers for each of the three SBE scenarios. Every student was asked to drop their surveys (answered or unanswered) into a box in the hallway outside the room. The survey contained five open-ended questions, including reasons for studying SAN and experiences (thoughts and feelings) with the attended SBE scenario (Supplementary Material 1). All data were treated as confidential, and personal data were used for demographic purposes only (Table 1). The facilitators left the immediate area during data collection. They collected the survey collection box for safe storage after the SAN students left the simulation centre. In sum, 93 of 105 (missing: n = 12) unique survey forms were collected for further analysis. Eight (n = 8) missing surveys were accounted for due to a misunderstanding in the handout process on one occasion.

### Data analysis

The data were analysed through inductive content analysis [30] by the first and last authors. The material was read and coded into group statements that described similar aspects, forming subthemes. The subthemes were reviewed, and related ones were grouped to form a theme. The summary of the analysis was reviewed and discussed by the three authors; any issues were resolved and a consensus was reached. The analysis resulted in two themes and four subthemes.

## Results

The results are based on the survey answers of 35 SAN students (their demographics are listed in Table 2).

**Table 2:** Demographics of the participants

Categories Groups	Age (years)			Gender	Experience as a R.N (years)			Experience A.N (years)		
	Range	Mean	SD		Range	Mean	SD	Range	Mean	SD
Total (n = 35)	25–50	33	6.02	Male = 20	1–26	6.69	4.5	0–8	1,98	2,10
				Female = 15						
Age groups										
< 30 (n = 12)	25–30	28.3	1.42	M = 4, F = 8	1–6	3.9	1.52	0–5	1.25	1.77
31–40 (n = 18)	31–40	34.2	3.26	M = 11, F = 7	3–16	7.3	2.93	0–8	2.30	1.94
41> (n = 5)	41–50	44.8	3.63	M = 5, F = 0	3–26	11.2	9.03	0–8	2.60	3,20

*RN = Registered nurse; AN = Ambulance Nursing; SD = standard deviation

In summary, participating in SBE during the programme provides a realistic understanding of the future profession and its expected demands. Furthermore, the SBE is perceived as a safe place that allows faulty decisions, as no one’s safety is compromised. Thus, all participants can focus on training, learning and personal development. The learning and development experience disregards prior work experience in ambulance services. The findings are presented in two themes: *SBE as learning* and *SBE as an educational method*. These themes are further divided into four subthemes (Table 3).

**Table 3:** Themes and subthemes of findings

Theme	Subtheme
SBE as learning	Improved understanding of the work and responsibilities
	Improved trust and insight into one’s ability
SBE as an educational method	Acting and processing the SBE
	Experienced facilitators’ ability to instil trust in one’s abilities

### SBE as learning

SBE as learning illustrates how a new professional role develops through SBE and facilitates gaining an understanding of future work. Furthermore, SBE allows students to understand their areas of development and instils confidence in one’s ability to comprehend and resolve a situation.

#### Improved understanding of the work and responsibilities

During the SBE, the students reported gaining insight into their future profession. They recognised that they could help in more ways than only from a medical perspective. They learned about the variety of patients that could be encountered, the living conditions of patients with a lower socioeconomic status and the different problems that could arise with patients, relatives or guardians when facing conflicts of interest. One student stated:

‘The simulation was authentic and provides insight into the fieldwork and the demands on the ambulance services to master difficult situations.’ (P2).

With the planning, support and execution during the SBE, the students also expressed an improved understanding of the impact of cooperation with colleagues on an ambulance mission. They considered communication to be the key component of ambulance care. This insight can also cause anxiety about working with other students as colleagues in the future, as one student mentioned:

‘A fear of having to work with some colleagues in the future…’ (P25).

In addition, they learned that they cannot prepare or be prepared for every situation. This understanding is expressed as the confidence to remain calm following this notion and that they can resolve situations with proper communication and ask for assistance. SBE enabled students to recognise the importance of feedback from colleagues and other healthcare personnel to improve and develop their abilities. As one student reported:

‘I take with me the importance of having an open discussion with my colleagues to express feelings and thoughts. . especially in a casual way, to double-check different alternatives and ideas.’ (P9).

Having a structured work process was also described as a key element. Thus, conducting a structured patient assessment helps to detect and manage potential problems. A clear communication structure also aids in cooperation with immediate colleagues, other colleagues and healthcare personnel. Finally, the SBE also created certain concerns that may be related to poor cooperation with colleagues, situations perceived as highly complex or a realisation that, in the future, a prolonged time might be spent with a critically ill patient before assistance arrives. One of the students stated:

‘I realised now that you actually could be spending a lot of time. . or be stuck. . at the scene with a really seriously ill patient before assistance could arrive. I need to make better use of bystanders and be able to communicate clearly and be structured with those around me.’ (P35).

#### Improved trust and insight into one’s ability

SBE can provide one with insight into areas in which knowledge deficits exist and improvement is needed. This notion is based on the understanding that solutions to a specific situation are not always definitive answers. Furthermore, SBE creates an understanding of the conditions that can be treated within ambulance care versus those that have heavily restricted treatment possibilities. One can also learn from SBE that clear and concise communication and planning, both before and during the scenario, could improve outcomes and performance. In addition, the students acknowledged that using and accessing different guidelines during patient encounters is allowed rather than trying to keep all expertise in one’s mind. Room for improvement in other aspects during the scenarios is always available:

‘The simulations created a lot of thoughts in me regarding how I want to work and what aspects I need to practice and learn more about. It also provides me with a sense of security in my upcoming profession.’ (P32).

During an SBE session, one’s limitations and what may need improvement were considered. SBE raises motivations to gain further knowledge, mostly because of one’s interest rather than from a medical perspective or due to the demands of the educational programme. Finding one’s work process structure and communicating clearly with others are important aspects. In addition, skills in reading the environment and collecting information from patients and bystanders should be improved. Furthermore, descriptions are provided of how one successively gains trust in the new professional role. The students boost their confidence in taking on a leadership role in different situations, although further development is also needed.

In the debriefing session, the students could gain feedback on how their appearance towards the patient or bystanders could be perceived. This feedback is described as an aspect one would need to reflect on and improve for future patient encounters:

‘I did not realise how my behaviour could be perceived by others; this was something that the facilitators could assist in reflection during debriefing. It feels good to practice, receive feedback and improve in future simulations or clinical practice.’ (P32).

### SBE as an educational method

SBE as an educational method illustrates the students’ views of SBE as an educational method and its possibilities and obstacles. In addition, this theme presents the important role of committed facilitators and structured preparations for the execution of SBE.

#### Act and process of SBE

One’s ability to immerse in SBE differs from feeling it as realistic. Factors hindering immersion are primarily related to technical difficulties with malfunctioning simulators and delays in audio transfer:

‘It is disabling and boring with the failures in equipment and technical obstructions.’ (P9).

Immersing oneself in SBE is easy when a facilitator plays the patient. In addition, the opportunity to perform twice in a similar scenario is perceived positively. This method provides one with the opportunity to adapt their way of work, evaluate it and observe any improvements directly. Furthermore, SBE is developmental for students, regardless of their experience in ambulance care, as even the smallest adjustments in cases could put additional stress on a student with experience. Thus, SBE exercises have no proper or improper rules; rather, they change according to choices made and over time.

‘It felt valuable even for one with several years of experience in the field.’ (P1).

SBE is described as a safe method, as no one is at risk of being hurt. Participants are allowed to make faulty decisions, as this contributes to learning. Moreover, SBE creates negative emotions, such as frustration and inadequacy, that build on one’s belief that he/she cannot perform. Nevertheless, these feelings are also based on insight that SANs are in an exposed situation when they have insufficient additional resources to help them or that specific patient groups in society are fragile. Feelings during SBE are also ambiguous; at times, one reflects on how he/she should feel during a certain situation. SBE can create numerous unexpected feelings:

‘I am relieved that it’s over and that it went well in the end. It is a bit surprising and funny that you can feel all these feelings during simulations, which are not real.’ (P3).

SBE also creates a solid foundation for further reflection in the subsequent debriefing. Students and facilitators can raise points of discussion that are appreciated by the students. In the debriefing session, the students were allowed to express in words their thoughts and feelings that emerged during the SBE while enhancing specific skills and knowledge related to the scenario. Students stated that they would prefer to have additional personal feedback, rather than feedback focused on the team and scenario and acknowledgement of whether they passed the SBE. Finally, the students described that they would prefer additional SBE during the programme. They believed that they developed the most during these sessions, as one student reported:

‘I actually enjoy the simulation sessions that put us in difficult situations with several alternative solutions. It gives the space for development in the following discussions.’ (P4).

#### Experienced facilitators’ ability to instil trust in one’s abilities

The facilitators conducted the SBE sessions with satisfactory planning and execution. The educational materials were acknowledged as preparations for the SBE days. Furthermore, the SBE days were perceived as clearly planned and structured. The facilitators were experienced and supportive, instilled trust in the students and highlighted the students’ strengths during the SBE. In addition, the facilitators were perceived as committed to the SBE. This trait improved the sense of realism in the scenarios, and the commitment was maintained in the subsequent debriefing session:

‘The simulation cases were good! Nice, sympathetic and experienced facilitators made these sessions structured, and the days passed quickly.’ (P15).

## Discussion

The findings show that the use of SBE in the programme is a challenging but safe way for both novice and experienced RNs to develop their knowledge and understanding of their future profession. A key aspect for successful SBE is motivated and enthusiastic facilitators with an interest and knowledge in ambulance nursing.

### SBE as an educational method – a tool for efficient learning

The findings suggest that SBE as a pedagogical tool appeals to students regardless of the extent of their experience in ambulance care, because they gain awareness of their abilities and approaches based on numerous scenarios. In addition, they acknowledge their need for development.

SBE is one of the many learning strategies available for educators and is suitable for groups of students who vary in experience, from novice to expert, and preferably adult students [31, 32]. During SBE, the student needs to be an active participant, either as a performer or as an observer, as both participation modes provide equal opportunity to construct knowledge [33]. Learning improves on an individual level, provides a unique experience and motivation and can be adjusted and improved as the student takes responsibility for their progression. Therefore, SBE is a valuable tool for individual learning for students and for a formative evaluation of the knowledge gained [31]. Formative evaluation refers to constructive feedback and assessment of the student’s progression relative to the course objectives [34]. In addition, SBE aimed at caring has a strong pedagogical influence, especially within the psychomotor area. However, using numerous learning activities remains important for meeting all educational objectives [35].

The findings also show that students learned how to be significant for patients in ways other than those based on medical perspectives. The SBE increased their understanding of collaboration with colleagues and others they encountered. SBE has also been found to strengthen reflective communication, stimulate empathy and contribute to an interprofessional learning community while nurturing interpersonal relationships [36]. Furthermore, communication and mutual reflection with colleagues and collaborative partners are key components in ambulance nursing and for professional and personal growth and learning through one’s and others’ experiences [37]. SBE could create a foundation for this component. Hence, SBE should focus not only on both technical and non-technical skills. Team training (i.e., communication, complicity, coordination and leadership) can be simulated and can provide participants with an experience of ‘critical competencies’. This training is most effective when conducted in a specific context (i.e. a patient suffering from a stroke and assessed and treated in an ambulance context) [31]. Moreover, learning is increased when simulated scenarios have emotional content [38]. In addition, teamwork is a key to patient safety and is thus an important part to be incorporated into education. In particular, as healthcare today is multidisciplinary, the interactions with colleagues with other backgrounds and experiences could influence patient care [37, 39].

In the current study, the students shared that they learned from each other through reflection during the debriefing session. They also considered this learning to be a part of the SBE where they learned the most efficiently. This statement is well documented and supported by the INACSL [40]. Reflection also becomes an important component to maintain in future professional work within ambulance nursing [37, 41]. Motola et al. [31] present that feedback provided by either facilitators or fellow students should preferably be on point and valuable for professional development. The most common form of feedback is formalised debriefing, which contributes to learning from one’s experiences in a reflective process. The feedback should be constructive and focused, rather than judgemental, and should allow students to improve. Debriefing is important for efficient learning and should be based on the students’ individual knowledge and abilities.

Being assessed and evaluated by facilitators and fellow students did not hinder the students from immersing themselves in the SBE in the current study. However, previous studies have presented otherwise, as students often experienced adverse feelings, such as stress, anxiety and fear, regarding the SBE and evaluation thereof [41, 42]. Based on previous research, when the SBE was adjusted to the individual level of knowledge and experience possessed by the students, SBE enabled them to make decisions, thereby facilitating learning [43].

### SBE as an educational method – the role of the facilitator

The students experienced the SBE as well planned and structured. Successful SBE requires careful planning (clear learning objectives, construction of a relevant scenario), pre-briefing (rules, prerequisites and expectations should be communicated) and debriefing (participants’ reactions, analysis or reflection, and knowledge gained). A further requirement is to evaluate the participants’ experiences and develop the scenario accordingly, if needed [32, 44].

The facilitators in the current study were supportive and instilled trust in the students. They highlighted the strengths as well as areas of improvement. Motola et al. [31] indicate that the facilitator’s role is to meet students on a suitable level of difficulty and provide feedback based on their knowledge and experiences. Jiménez-Gómez et al. [45] claim that reflective and critical thinking are central aspects of nursing education. They advocate for problem-based learning to develop problem-solving, decision-making and communication skills, as well as analytical skills for one’s actions. Educating facilitators is crucial to gain conformity between pedagogical models (didactic strategies), teaching strategies and evaluations [46]. Furthermore, the facilitators in the current study had extensive experience in the clinical context of ambulance nursing. The students acknowledged this aspect, which provided additional realism to the simulated scenarios. In addition, scenarios with varying and relevant patient encounters are important for educating competent care providers professionally. SBE allowed the students to be taught how to handle unexpected situations and emergencies without jeopardising their own safety or that of the patients [31]. An interesting future research avenue would be to investigate the strategies utilised by facilitators to ensure successful SBE.

### SBE as learning – the preparation for a new professional role

In this study describing SAN students’ learning experience in SBE, the students’ demographics were used to contrast experiences. However, the experiences did not differ, because the SAN students considered SBE to be a measure for preparation for a new professional role. This result is intriguing, as the demographics in the study revealed diverse experiences as an RN and in the context of ambulance nursing.

The participants were also asked to describe their motivational factors for applying to and undertaking this education. The motivational factors were both internal and external. Striving to expand one’s knowledge and skills in the specialist area was the primary internal factor, whereas the external factors included formal competence, along with increased salary and demands from ambulance organisations for employment. Medical students described similar motivational factors, where altruism, personal satisfaction, economic feedback and gaining personal and supportive social networks and a diploma were reported [47].

The present SBE appealed to this diverse group of students regarding experience and motivational factors to complete their education. However, some potential challenges could be encountered related to conducting SBE with participants who differ in both clinical and SBE experience. One is the presence of relevant and achievable objectives and goals in the SBE [48]. Another is acknowledging that older students may never have experienced SBE as a learning activity; therefore, the nurse educator requires an open and responsive state of mind in relation to the students’ learning needs [49]. Vital aspects include continuous work for the creation of psychological safety (i.e. making mistakes without consequences), the qualities of the facilitator and orientation activities when evaluating and developing SBE [50]. In this study, the facilitators who planned the SBE were RNs with extensive experience in ambulance or emergency care and SBE didactics. They also used the INACSL standards of best practice [27] as a guide when designing the course.

### Limitations

Numerous limitations must be considered in this study. Since one of the authors is employed full-time in the current SAN programme, the analysis was done by the other two authors. One was never involved in teaching the group of SAN students, while the other had only sporadic interactions with them. This division is believed to contribute to maintaining student integrity by preventing the analysis of their responses by an examination facilitator. Furthermore, as the authors have previous experience in SBE in various nursing programmes at the University, they might have a preunderstanding, which could influence the interpretation of the results. Conversely, the authors were interested in gathering feedback that could develop their SBE; therefore, they actively sought aspects related to positive and negative critiques. In addition, the findings were discussed with three senior researchers who were not involved in the research process as a way to obtain opinions related to unbiased descriptions of the findings and to question potential preunderstandings. The participants’ responses could also be influenced and they may only express their positive experiences or express what they think their facilitators want to hear, thus resulting in social-desirability bias and a potential misleading result in the end [51]. However, negative critiques regarding the SBE experiences were also received, though mostly aimed at different technical and mechanical malfunctions. Furthermore, the participants are vulnerable to their facilitators, which may influence their decision to participate in this study. This limitation has been addressed by providing them with information weeks before the study participation, at the start of the SBE, and again after the SBE scenarios and by allowing them to choose, unattended, whether to answer the paper-based survey. Furthermore, the responses varied in detail and scope due to free text answers. More detailed results could have been generated if other methods, such as interviews, had been used. Therefore, conducting a similar study using interviews with participants after performing the SBE would be interesting. This approach would also allow asking the participants to answer follow-up questions and to provide detailed examples of their described experiences. Considering that this study had 35 participants, the authors do not believe that a larger number of participants would have influenced the results in any significant way, since the results present both positive and negative aspects of SBE.

## Conclusions

This study shows that a well-planned SBE with carefully selected scenarios is a recognised educational method regardless of experience in the field of prehospital care or as an RN. Even if SBE is an expensive form of pedagogical activity, it seems to be an appreciated method among students. This appeal could assist in the retention of students in various post-graduate nursing education programmes. Furthermore, SBE provides a foundation for future work as a SAN and stimulates students regardless of their prior experience in ambulance care. Future research should aim at further exploration of the strategies used by SBE facilitators in post-graduate nursing educational programmes to generate successful learning opportunities for students with diverse experience. In addition, future research can also aim to gain in-depth descriptions of the students’ experiences with SBE and to further investigate the aspects that are learned and whether these learning outcomes are transferred to the professional role in clinical practice.

### Electronic supplementary material

Below is the link to the electronic supplementary material.

Supplementary Material 1 - Survey (English version)

## Data Availability

The dataset analysed during the current study is available from the corresponding author upon reasonable request. Be advised that the data are in Swedish and, therefore, might be of limited support.

## Abbreviations

ECT: European Credit Transfer System
INACSL: International Nursing Association of Clinical and Simulation Learning
RN: Registered Nurse
SAN: Specialist Ambulance Nurse
SBE: Simulation–Based Education
