# Supplementary material for: Simulation-based education as a provider of fieldwork insights – experiences of ambulance nurse specialist students
Source: BMC Nurs. 2023 Dec 19;22:485. doi: 10.1186/s12912-023-01666-2 (PMC10729564; doi:10.1186/s12912-023-01666-2)
Supplement: Supplementary file 1 — Supplementary Material 1 - Survey (English version) [file 12912_2023_1666_MOESM1_ESM.docx]

**Supplementary material – Study form**

**Would you like to participate in a pilot study regarding your learning experience during the simulation towards fragile and vulnerable patients?**

This means that you fill out this form after each simulation scenario.

Yes  No

**A few background questions:**

Age:____ (years)

Experience as a registered nurse:____(years)

Experience from ambulance care:_____(years)

Sex: Male Female I don’t identify with either

What are your goals in undertaking this education?

____________________________________________________________________________________________________________________________________________________________________

**Which scenario did you take part in this occasion?**

Aa Ab Ba Bb Ca Cb

**What are your thoughts after this simulation?**

__________________________________________________________________________________________________________________________________________________________________________________________________________________________________________________________________________________________________________________________________________________________________________________________________________________________

**What are your feelings after this simulation?**

**__________________________________________________________________________________________________________________________________________________________________________________________________________________________________________________________________________________________________________________________________________________________________________________________________________________________**

**Are there anything you can take with you from the simulation today?**

__________________________________________________________________________________________________________________________________________________________________________________________________________________________________________________________________________________________________________________________________________________________________________________________________________________________

**Are there anything you would like to share from the simulation today?**

**__________________________________________________________________________________________________________________________________________________________________________________________________________________________________________________________________________________________________________________________________________________________________________________________________________________________**

*Your answers will be treated confidentially, and the pilot project will provide a foundation for an upcoming research study as well as the development of simulation as a pedagogical tool.*

**We really appreciate you participation!**

*enter names after review process XXXXXXXXXXXXXXXXXXX*
